# Supplementary material for: Synthesis and Characterization of Click Chemical Probes for Single-Cell Resolution Detection of Epichaperomes in Neurodegenerative Disorders
Source: Biomedicines. 2024 Jun 4;12(6):1252. doi: 10.3390/biomedicines12061252 (PMC11201208; doi:10.3390/biomedicines12061252)
Supplement: Supplementary file 1 [file biomedicines-12-01252-s001.zip › biomedicines-3024218-supplementary.pdf]

Supplementary Information

Bay et al.

Supplementary Figures S1-S4: Purity and identity characterization of the click probes, PU-TCO and the negative control PU-NTCO

Supplementary Figure S5: uncropped gels

a

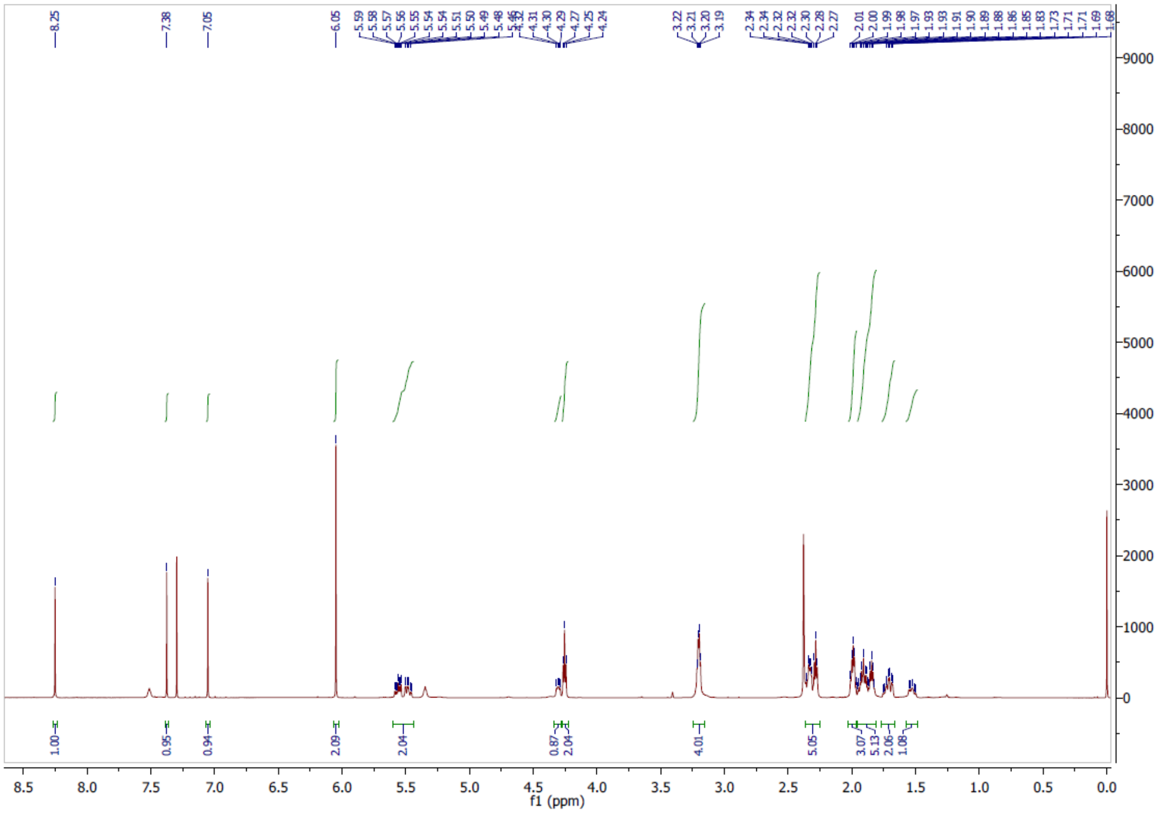

b

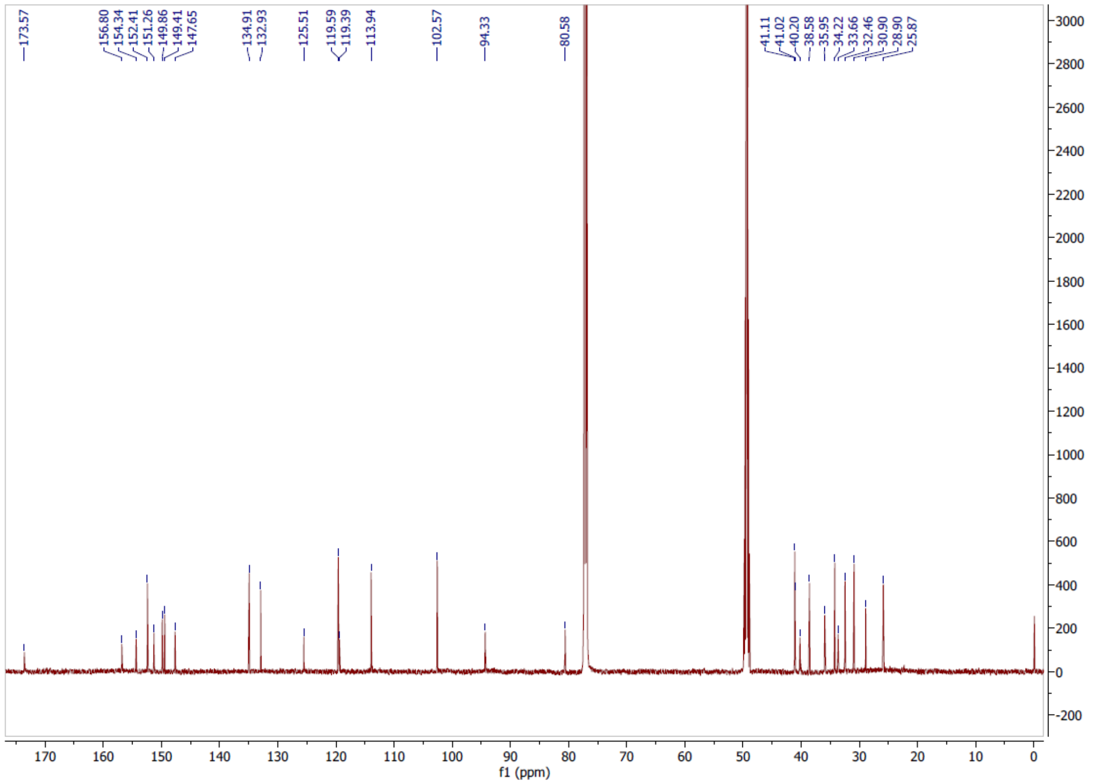

Supplementary Figure S1. Structural characterization of PU-TCO by nuclear magnetic resonance spectroscopy (NMR). a ^{1}H NMR spectra in $CDCl_3/CD_3OD$. **b** ^{13}C NMR spectra in $CDCl_3/CD_3OD$.

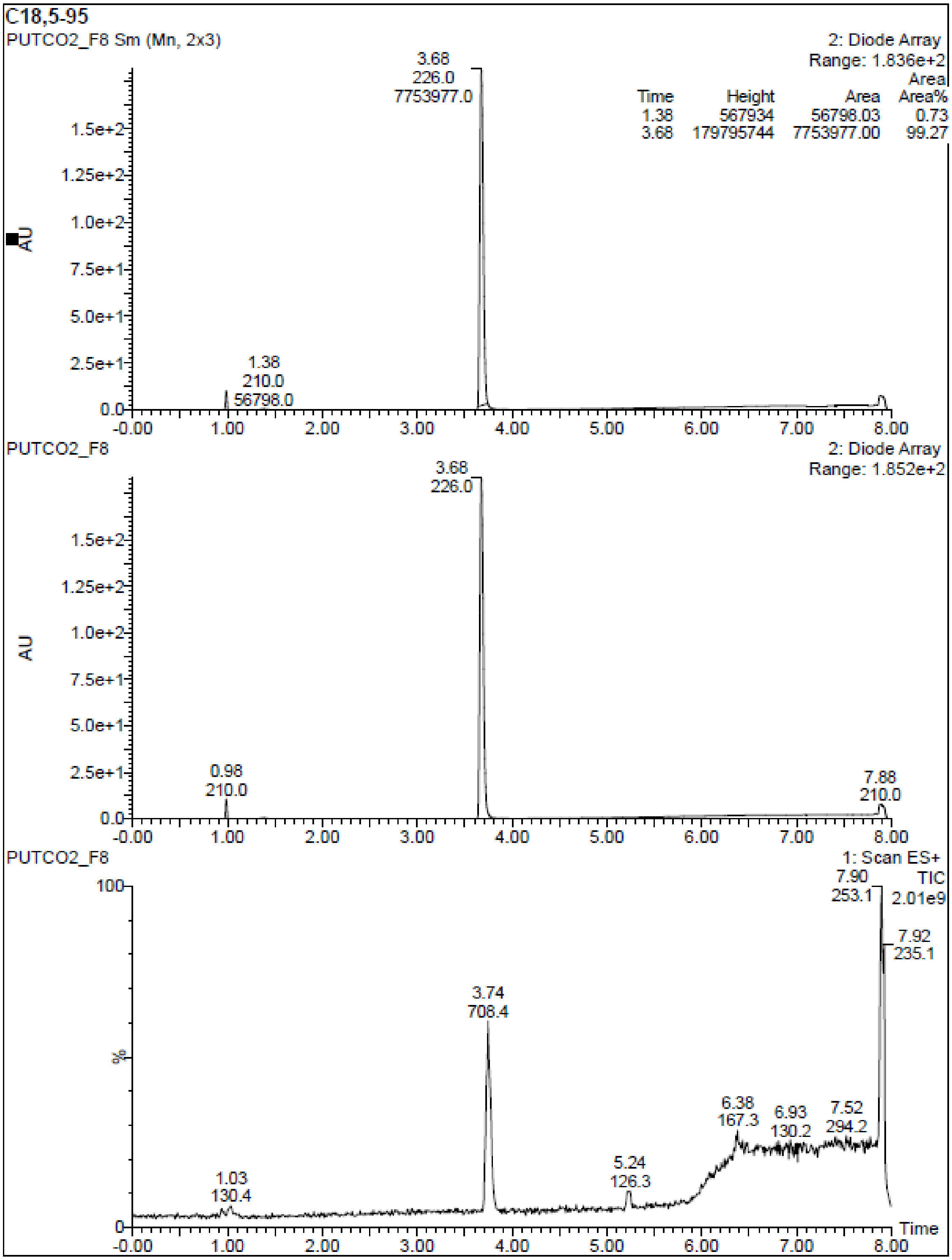

Supplementary Figure S2. Liquid chromatography-mass spectrometry (LC-MS) chromatogram of PU-TCO.

a

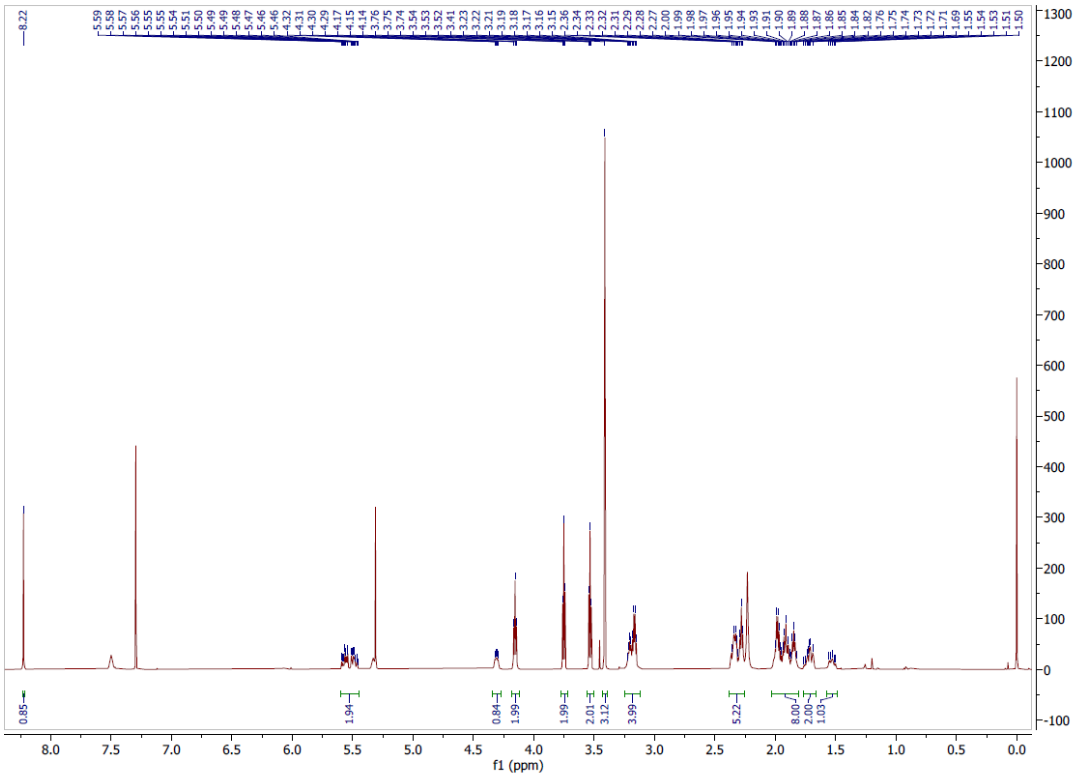

b

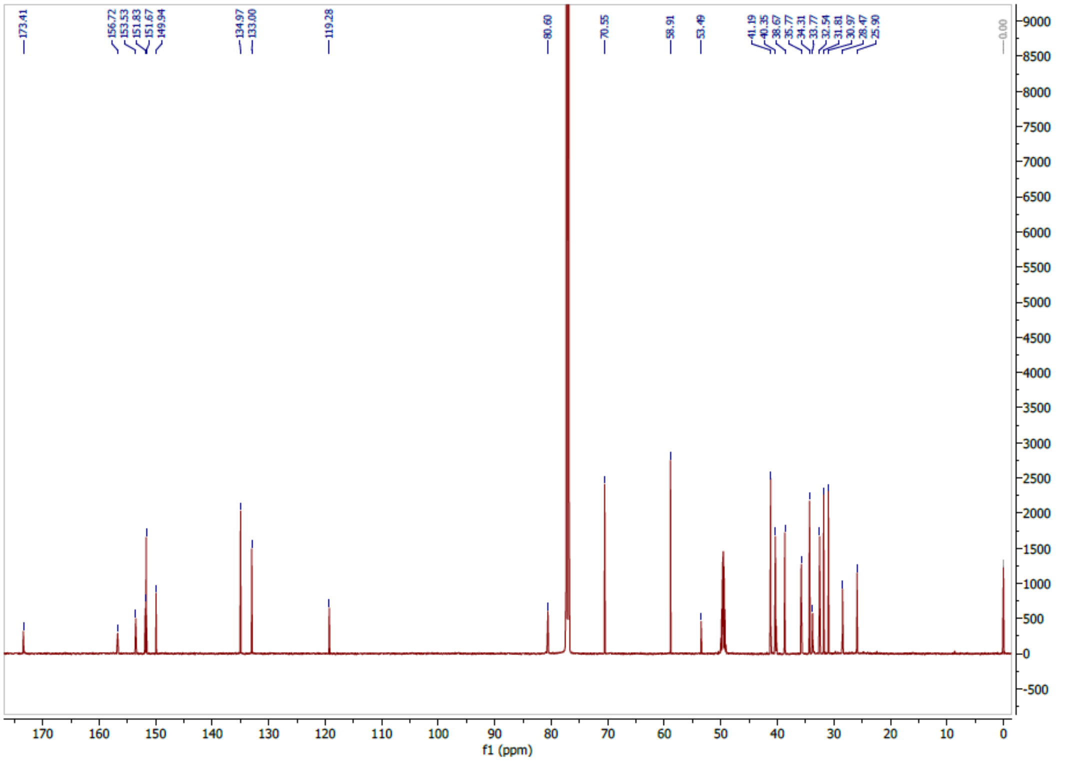

Supplementary Figure S3. Structural characterization of PU-NTCO by nuclear magnetic resonance spectroscopy. a ^{1}H NMR spectra in $CDCl_3/CD_3OD$. **b** ^{13}C NMR spectra in $CDCl_3/CD_3OD$.

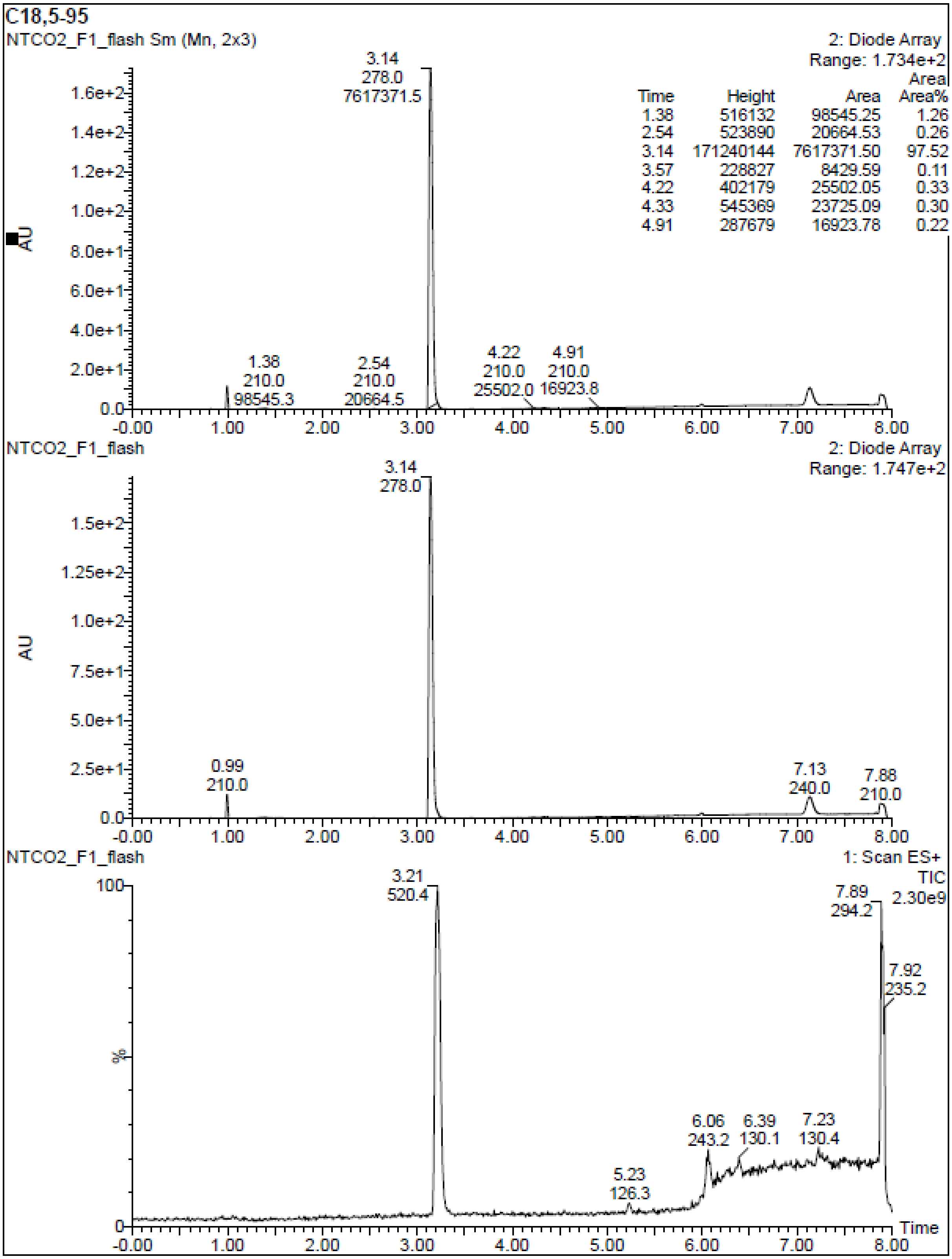

Supplementary Figure S4. LC-MS chromatogram of PU-NTCO.

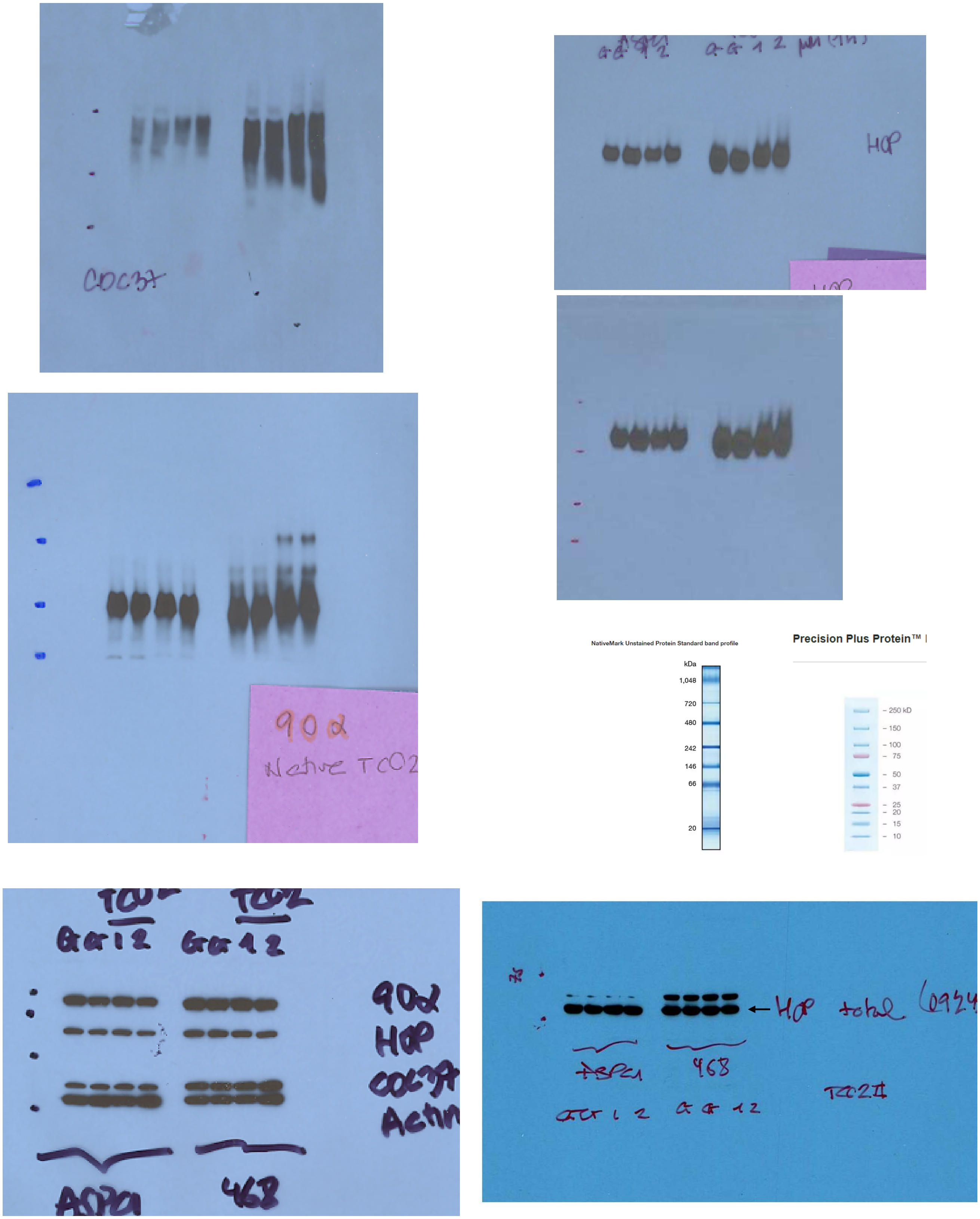

Supplementary Figure S5. Uncropped gels associated with Figure 3b and the molecular weight marker standards that were used for determining the approximate size of the proteins (Precision Plus Protein Marker) or protein assemblies (NativeMark Unstained Protein Standard) run on the electrophoresis gel.
